# Supplementary material for: Mortality causes universal changes in microbial community composition
Source: Nat Commun. 2019 May 9;10:2120. doi: 10.1038/s41467-019-09925-0 (PMC6509412; doi:10.1038/s41467-019-09925-0)
Supplement: Supplementary file 1 — Supplementary Information [file 41467_2019_9925_MOESM1_ESM.docx]

Supplementary Information for

Mortality causes universal changes in microbial community composition

Abreu et. al.

**Supplementary Figures**

**Supplementary Figure 1: In ten pairwise competitions, coexistence is more common than bistability.** All ten pairwise competitions between the five bacteria species are shown. The fraction of the faster grower is plotted on the y-axis. The title of each plot lists the faster grower first, and the “subway map” plots the faster grower above the slower grower. Gray arrows represent time trajectories, beginning at the starting fraction, and ending at the final fraction, five to seven days later. The solid black lines show the equilibrium fixed points, and dashed black lines show unstable fractions. Coexisting pairs have unstable fractions of one and/or zero, while bistable pairs may have unstable fractions between one and zero. Stable fractions of coexisting pairs were determined by averaging final fractions across all replicates. Error bars are the SEM across replicates. (n ≥ 4 for all pairs at all dilution factors. Each competition was reproduced at least two times. Most were reproduced at least three times.) In coexisting pairs, equilibrium fraction increases with dilution factor, showing that faster growers are favored by increased death (except in the case of *Pp*-*Ea*; these two species are the fastest growers and have the most similar estimated growth rates, with p = 0.08, compared to p < 0.001 for all other pairs of species). Additionally, in bistable pairs *Pci*-*Pv* and *Pp*-*Pv*, the unstable fraction shifts in favor of the faster grower as death increases. Some pairs, particularly those including species *Pa*, displayed high variability at equilibrium at intermediate dilution factors. However, most final fractions were coexisting, so those outcomes were categorized as coexistence. Such a simplification is necessary for categorization and making predictions about multispecies states, but does not fully explain the observed outcomes. In particular, some pairs show evidence of nontraditional bistability between exclusion of one species and coexistence of both (as opposed to bistability of exclusion of either species), such as *Pa*-*Pv* and *Pci*-*Pv* at dilution factor 100. This is an outcome not allowed by simple models, but overall it is not common, and it is not always reproducible. In some cases, variability is due to incomplete equilibriation or outcomes that shift slightly from experiment to experiment. We observe the traditional three outcomes (dominance, coexistence, bistability) allowed by simple models in ~90% of results.

**Supplementary Figure 2: Pairwise assembly rules make good quantitative predictions of three- and four-species states. a-b)** Data from two different three-species experiments is compared to predictions based on pairs and monocultures. Monoculture predictions use carrying capacities (Fig. S5-C,D) and modified monoculture predictions also incorporate dilution and growth (in the logistic model with added death, the carrying capacity is multiplied by a factor, $(1-\frac{\delta}{r})$). Pairwise predictions are based on the results shown in Fig. S1. Errors of quantitative predictions are the L2 norm of the distance between predicted fixed point and observed fixed point, with maximum error equal to $\sqrt{2}$. The values shown in the quantitative column are error/maximum error; values in the qualitative column are number of species correctly predicted as present/absent. **c)** Quantitative prediction errors for all three- and four-species experiments is shown. Accuracy was calculated the same way as in **a**-**b**. End-point results from multiple initial fractions of a given community were averaged before measuring error (in the case of bistability, data was separated and then averaged). The mean error plotted is the average of the means of three- and four-species predictions; error bars are propagated from the SEM of three- and four-species predictions (for four-species experiments, n=96 in defined medium, n=120 in complex medium; for three-species experiments, n=118 in defined medium, n=191 in complex medium). **d)** Qualitative accuracy for all three- and four-species experiments was calculated by comparing predicted species presence to results. A 3% relative abundance threshold was used to determine presence of a species.

**Supplementary Figure 3: Growth rates of single species were determined by measuring time to a threshold density.**  To measure growth rates, species were grown in monoculture, with optical density measured over a period of ~50 hours. Before these measurements, species were grown in 1X LB broth overnight, and then transferred to the experimental medium for 24 hours. The OD of all species was then equalized. The resulting cultures were diluted into fresh medium at factors of 10^-8^ to 10^-3^. One replicate of such an experiment is shown in **a**. The same data is shown in **b** on a log scale. In **c**, one set of growth curves is shown. Background has been subtracted, but in order to better interpret the data, curves were smoothed (using an exponentially weighted moving average, as well as by discarding low-OD noise), as shown in **d**. Plot **e** shows how the smoothed curves were then used to measure growth rates: a threshold OD of 0.1 was chosen, and exponential growth was assumed to occur until this threshold, as seen by arrows, which are drawn from starting OD to threshold OD. By assuming exponential growth to a threshold, we assume no lag time occurs, but the resulting measurement implicitly incorporates lag: longer lag times will cause the measured growth rate to be lower, while shorter lags will have the opposite effect. Final growth rate measurements were determined for each species by averaging these measurements across all replicates (n=21, per species). *Ea* and *Pp* had the most similar estimated growth rates (p = 0.08, compared to p < 0.001 for all other pairs of species).

**Supplementary Figure 4: Exponential growth rate, time lag estimated with time-to-threshold data.** To measure exponential growth rate and time lag, we plotted starting OD of monocultures against their time to the 0.1 OD threshold. Assuming a time lag $\tau$ of zero growth followed by exponential growth$r$ over a period of time $t_{t}$ to the threshold $OD_{t}$, we can write:

$$\begin{aligned} OD_{t}=OD_{o}e^{r\left( t_{t}-\tau\right)} \#\left( 1 \right) \end{aligned}$$

This equation can be re-arranged:

$$\begin{aligned} t_{t}-\tau= -\frac{1}{r}\ln OD_{o}+\frac{1}{r}\ln OD_{t} \#\left( 2 \right) \end{aligned}$$

We can thus plot the starting OD on the x axis, and the time taken to reach the threshold OD on the y axis. The slope of a regression line is our estimate of $-\frac{1}{r}$, and the time lag estimate can be found with the y-coordinate of the regression line where its x-coordinate equals the threshold OD.

**Supplementary Figure 5: Growth rate is weakly correlated with lag time.** There are different ways of measuring growth, and one might argue that our time-to-threshold method (Fig. S3) is inferior to explicitly measuring exponential growth rate and time lag. Plot **a** shows how threshold growth rate compares to exponential rate: rates are positively correlated but might switch order. We estimate effective growth rate by multiplying exponential rate by a function of lag and time between dilutions (supplementary section S4). As shown in **b**, this adjustment preserves the ordering of growth rates and shows a strong correlation. Plots **c** and **d** show that there is no correlation between growth rate and carrying capacity (large errors are due to absolute abundance being more variable than relative abundance in colony-counting assays). Plots **e** and **f** show that threshold growth rate has a weak negative correlation with time lag, but such a correlation is not significant when using exponential growth rates. This indicates that slower growers may have longer lags, especially because those lags are implicit in the threshold growth rates. This would seem to be disadvantageous at low dilution factors, where time lags prevent consumption of precious resources, making the dominance of slow growers at low dilution factors hard to explain. Error bars are the SEM of replicates for threshold growth rate (n=21, per species) and carrying capacity (n=2, where two measurements with three replicates each were averaged over three days of growth and saturation), and the SD from 5,000 bootstrap trials (using data in Fig. S4) for lag and exponential growth rate (n=21, per species). Slope statistics are from function lm in R.

**Supplementary Figure 6: Spent media of some slow growers can inhibit some fast growers, but ordering of growth rates does not switch at low resource concentrations.** In an attempt to explain the dominance of slow growers at low dilution factors, we performed two types of experiments. First, to test for presence of toxins or other compounds, all five species were grown in the other species’ spent media, as well as their own (**a-e**). Spent media was prepared by filtering saturated monocultures and then replenishing the carbon sources. In a 96-well plate, 10ul of 20X carbon sources were added to 190ul of supernatant (spent media) and cells of a particular species from two starting densities (all plots except right panels of **b** and **d**, which have three starting densities). Thus, the assay tested for antagonism by non-resource mediated mechanisms such as toxins that slow growers might use to dominate in high-density competition. None of the species were unable to grow, but *Pp* was inhibited by *Pa* supernatant (**b**; *Pa* is a slower grower) and *Pci* was inhibited by *Pv* supernatant (**d**; *Pv* is the slowest grower). *Pp* was also inhibited by *Pv* in one round of experiments, but this effect was not reproduced (**b**). Thus, non-resource mediated antagonism most likely explains some, but not all, of dominance of slow growers in low-dilution, high-density conditions. Second, to test for a tradeoff between growth rate and resource concentration, we grew all species in the experimental media with low concentrations (1/30X, 1/100X) of carbon sources (**f**). If such a tradeoff existed, it would appear as a flipped ordering of growth rates, in which slow growers become relatively fast growers at low resource concentrations. This could impart a competitive advantage in low-dilution environments, where cultures spend much of their daily cycles in saturated or nearly-saturated conditions with low amounts of resources. However, we did not observe such a flip in growth rate ordering. Error bars are the SEM of replicates (n=3).

**Supplementary Figure 7: Competitive scores in complex medium follow a similar pattern to that in defined medium.** Coculture experiments were also performed with four species in a complex and undefined medium: 0.1X LB broth. The order of growth rates in this medium is similar to that in the defined medium (Fig. 4). Additionally, the competitive scores at low dilution factor are negatively correlated with growth rate; as dilution increases, competitive score becomes positively correlated with growth rate. Results in the undefined medium are less reproducible than in the defined medium, because ingredients may change slightly from batch to batch, but the negative correlation between competitive score and growth rate at low dilution factor is reproducible. (Our pairwise results in complex media were good predictors of multispecies states (Fig. S2) because both types of experiments were performed using the same batch of LB broth.) Error bars for growth rates are the SEM of replicates (n=10, per species); the error bars for competitive score are were calculated by bootstrapping, where replicates of mean experimental outcomes of a given pair were sampled 5,000 times with replacement (n=31, per species, per dilution factor).

**Supplementary Figure 8: Range of dilution factors over which species survive can vary between experiments.** The remaining plots from Fig. 1 are shown (**a-b**). The coculture experiments began with 90% of each species (3 replicates) as well as equal amounts of the three species (final replicate). This experiment was repeated two more times (**c-d**) with slightly different results. The structure of the subway map is not significantly changed (**c**), but some states became stable that weren’t stable before, and vice versa. For example, *Ea*/*Pci* becomes a stable state at DF 10^2^ when co-cultures begin with 95% of *Ea* or *Pci*, as in experiments 2 and 3. The stability of *Ea* at DF 10 and *Pci* at DF 10^5^ varied between experiments, both of which are dilution factors adjacent to those where these species are usually stable. Error bars are the SD of the beta distribution with Bayes' prior probability (see Methods).

**Supplementary Figure 9: Accuracy of assembly rules decreases with number of species due to slow equilibriation and infrequent coexistence of more than two species.** Results of additional three- (**a-b**), four- (**c**), and five-species (**d**) coculture experiments are shown. Each starting fraction is plotted separately for a given experiment. The subway maps predicted by the pairwise assembly rules are shown above the results. In the trios, species appear or do not appear in states as predicted at a rate of about 90%. For example, the first starting fraction of Trio **a** fulfills 3/3 = 100% of species predictions, while the third fulfills 2/3 = 67%. Trio **b** has a higher success rate than Trio **a**, in part because only two species are predicted to coexist in any given state, while three species are predicted to coexist in many of Trio **a** states. Coexistence of more than two species does not appear consistently across multispecies experiments, perhaps due to the limited number of carbon sources in the medium. In this study, coexistence of all three species in a trio occurred ~25% of the time it was predicted in the defined medium. In the complex medium (not shown here), three-species coexistence predictions were more accurate, with a success rate of ~75%. In the four- and five-species communities (**c-d**), prediction success further decreases, perhaps because: equilibriation is slower with more species, predictions of multispecies coexistence fail, or starting conditions cause rare species to go extinct before establishing.

**Supplementary Figure 10: Phylogenetic tree of the set of five species used in this study.** The tree is based on the full 16S gene and the branch lengths indicate the number of substitutions per base pair.

| Prediction Type | Mean error / max error, 3-species communities | Mean error / max error, 4-species communities | Mean error / max error, 5-species community |
| --- | --- | --- | --- |
| Pairwise Outcomes | 0.09 (0.02) | 0.19 (0.02) | 0.31 (0.05) |
| Modified Monocultures | 0.30 (0.03) | 0.35 (0.04) | 0.30 (0.08) |
| Monocultures | 0.40 (0.03) | 0.43 (0.03) | 0.36 (0.08) |
| Random Predictions | 0.46 (0.03) | 0.47 (0.03) | 0.43 (0.06) |

**Supplementary Table 1: Accuracy of assembly rules decreases with number of species.** An extension of Table 1 from the main text shows all prediction data for three-, four- and five-species experiments in the defined medium. While the assembly rules are the best predictor of three- and four-species states, they do not offer improved accuracy in the case of the five-species community. Mean errors of three types of predictions are shown, as well as mean error for random predictions, for comparison. Monoculture predictions use carrying capacities (Fig. S5-C,D) and modified monoculture predictions also incorporate dilution and growth (in the logistic model with added death, the carrying capacity is multiplied by a factor, $(1-\frac{\delta}{r})$). Pairwise predictions are based on the results shown in Fig. S1. Errors of quantitative predictions are the L2 norm of the distance between predicted fixed point and observed fixed point (see Methods and Supplementary Figure 2). The values shown are mean error normalized by the maximum error. Errors, shown in parentheses, are SEM of replicates (n=118 for 3-species communities, n=96 for 4-species communities; n=26 for the 5-species community; outcomes of different starting fractions of the same biological replicate were averaged before measuring error, and in the case of bistability, the smaller of the two errors was chosen after averaging outcomes from different starting fractions ending at the same final state).

**Supplementary Discussion**

**Supplementary Note 1: Derivation of Lotka-Volterra model modified by added death**

The most basic form of the two-species Lotka-Volterra model takes the following form:

$$\begin{aligned} \frac{\dot{N_{i}}}{N_{i}}= r_{i}-c_{ii}N_{i}-\sum_{j} c_{ij}N_{j}\#\left( 3 \right) \end{aligned}$$

where $r_{i}$ is the exponential growth rate of species $i$ (minus any intrinsic death rate), $c_{ii}$ is the rate at which species $i$ inhibits itself, and $c_{ij}$ is the rate at which species $j$ inhibits species $i$. Equation (3) can be re-parameterized to:

$$\begin{aligned} \frac{\dot{N_{i}}}{N_{i}}= r_{i}\left( 1-\frac{N_{i}-\sum_{j} \beta_{ij}N_{j}}{K_{i}} \right)\#\left( 4 \right) \end{aligned}$$

where $K_{i} =\frac{r_{i}}{c_{ii}}$ is the carrying capacity and $\beta_{ij} =\frac{c_{ij}}{cii}$ is the competition coefficient. We can further re-parameterize the model by normalizing by carrying capacity:

$$\begin{aligned} \frac{\dot{\hat{N_{i}}}}{\hat{N_{i}}}= r_{i}\left( 1-\hat{N_{i}}-\sum_{j} \alpha_{ij} \hat{N_{j}} \right)\#\left( 5 \right) \end{aligned}$$
where $\hat{N_{i}}=\frac{N_{i}}{K_{i}}$ and $\alpha_{ij}=\beta_{ij}(\frac{K_{j}}{K_{i}})$. This version of the model is useful because the competition outcomes depend upon whether the competition coefficients are greater or less than one: stable coexistence occurs when both coefficients are less than one, bistability when both are greater than one, and dominance/exclusion when only one coefficient is greater than one. This leads to the log/log phase space (Fig. 2c, again in Supplementary Fig. 11), in which boundaries form where competition coefficients equal one.

The modified Lotka-Volterra model includes an added global death term:

**Supplementary Figure 11:** Re-parameterization of model allows for division of phase space where competition coefficients equal one.

$$\begin{aligned} \frac{\dot{\hat{N_{i}}}}{\hat{N_{i}}}= r_{i}\left( 1-\hat{N_{i}}-\sum_{j} \alpha_{ij} \hat{N_{j}} \right)-\delta\#\left( 6 \right) \end{aligned}$$

This term can be absorbed in order to return the model to its previous form (Equation (5)):

$$\begin{aligned} \frac{\dot{\tilde{N}_{i}}}{\tilde{N}_{i}}= \tilde{r}_{i}\left( 1-\tilde{N}_{i}-\sum_{j} \tilde{\alpha}_{ij}\tilde{N}_{j} \right) \#\left( 7 \right) \end{aligned}$$

where $\tilde{r}_{i}=r_{i}-\delta,$ $\tilde{N}_{i}$= $\frac{\hat{N_{i}}}{1-\frac{\delta}{r_{i}}}$ and $\tilde{\alpha}_{ij}=\alpha_{ij}\frac{1-\frac{\delta}{r_{j}}}{1-\frac{\delta}{r_{i}}}$. Multiplying $\alpha_{ij}$ by a term means that we add a term to ${log \alpha}_{ij}$. Due to symmetry, the same term will be subtracted from $\log\alpha_{ji}$. As a result, increasing death causes the outcome to move in a line with a slope of negative one through the log/log phase space (Supplementary Figure 11), beginning at the outcome with no added death, (${log \alpha}_{ij}$, ${log \alpha}_{ji}$). If this outcome resides in the quadrant where the slow grower wins, increasing death will eventually result in the fast grower winning. If the trajectory begins in the quadrant where the fast grower wins, however, increasing death will not change the outcome.

While a global death rate of $\delta$ leads to the simple prediction that the fast grower is favored, it is not the most realistic scenario. In reality, different species may be affected by different added death rates. In this case, the expression for the competition coefficients becomes:

$$\begin{aligned} \tilde{\alpha}_{ij}=\alpha_{ij}\frac{1-\frac{\delta_{j}}{r_{j}}}{1-\frac{\delta_{i}}{r_{i}}} \#\left( 8 \right) \end{aligned}$$

Taking the log of Equation (8) results in addition of a term to ${log \alpha}_{ij}$, the same term which will be subtracted from ${log \alpha}_{ji}$. The outcomes will therefore still move along the same 45° line through the phase space, although not necessarily at the same rate or in the same direction. Added mortality will favor the faster grower if the following condition is met:

$$\begin{aligned} \frac{\delta_{s}}{\delta_{f}}>\frac{r_{s}}{r_{f}} \#\left( 9 \right) \end{aligned}$$

We therefore see that the fast grower can still be favored if it is killed at a higher rate (as in the case of $\beta$-lactam antibiotics, which target faster growers by inhibiting cell wall biosynthesis). Furthermore, the growth/competition tradeoff at low dilution is not required to observe outcome changes if the slow grower is selectively targeted; in this case, the trajectory would move from fast grower winning at low mortality, to coexistence or bistability at intermediate mortality, to the slow grower winning at high mortality.

**Supplementary Note 2: Lotka-Volterra model outcomes are the same with discrete, continuous dilution**

To make qualitative predictions about how competition outcomes will change as dilution rate increases, we have used the Lotka-Volterra (LV) model with an added global death rate. We use discrete (not continuous) dilutions in laboratory experiments, however, which raises the question of whether the continuous model’s predictions apply to our experiments. In fact, a discrete death rate can be converted to an equivalent continuous death rate in the model. While the time dynamics of the system will differ, the outcome will be the same under both types of death rate.

To show this, we first note that the per-capita growth rate in the LV model is linear and additive:

$$\begin{aligned} \frac{\dot{x}_{i}}{x_{i}}=r_{i}\left( 1-\sum_{j}^{N} \alpha_{ij}x_{j} \right)-\delta\#\left( 10 \right) \end{aligned}$$

These properties ensure that the model can be time-averaged without losing its form (addivitity refers to the fact that no covariance results from averaging). The outcome of the time-averaged model will be the same as if the variables and parameters were replaced by their time-averages (Fox, 2013):

$$\begin{aligned} \left\langle\frac{\dot{x}_{i}}{x_{i}} \right\rangle=r_{i}\left( 1-\sum_{j}^{N} \alpha_{ij}\left\langle x_{j} \right\rangle\right)-\left\langle\delta\right\rangle\#\left( 11 \right) \end{aligned}$$

In other words, the model predicts that an oscillating death rate can be replaced by its time average. A discrete daily dilution is effectively an oscillating death rate, in which the death rate oscillates between zero and an abrupt removal of much of the population. We can model this removal as exponential death at rate $\delta$ over a very short period of time $t$:

$$\begin{aligned} N_{f}=N_{i}e^{-\delta t} \#\left( 12 \right) \end{aligned}$$

In fact, we are dividing the population size by the dilution factor $DF$. In the limit that $t$goes to zero and $\delta$goes to infinity while their product is constant, exponential death is equivalent to division by a dilution factor. We can equate the two results and solve for the effective exponential death rate:

$$\begin{aligned} N_{i}e^{-\delta t}=\frac{N_{i}}{DF} \#\left( 13 \right) \end{aligned}$$

$$\begin{aligned} \delta=\frac{\ln DF}{t} \#\left( 14 \right) \end{aligned}$$

For the rest of the cycle, which has length $T$, death rate is zero. Thus we can average the two rates by weighting them appropriately:

$$\begin{aligned} \left\langle\delta\right\rangle= 0*\frac{T-t}{T}+\frac{\ln DF}{t}*\frac{t}{T}=\frac{\ln DF}{T} \#\left( 15 \right) \end{aligned}$$

The equivalent continuous death rate to a discrete dilution is thus the natural log of the dilution factor divided by the time between dilutions. In the model, both discrete and continuous rates yield the same outcome.

**Supplementary Note 3: Time lag and exponential growth rate can be combined to form effective growth rate**

The LV model predicts that an added death rate will favor the faster of two species in a pairwise competition. But if growth rates are similar enough, other factors such as time lag may become more important.

Time lags can affect competition outcomes. Daily dilution experiments allow for the existence of lags, because cultures are periodically diluted, often from the point of saturation. One option for treating a lag is to incorporate it into growth rate. Assuming a lag of zero growth lasting time $\tau$, followed by exponential growth for the remainder of the cycle of time $T$, a population will grow as follows:

$$\begin{aligned} N_{f}=N_{i}e^{r\left( T-\tau\right)}=N_{i}e^{r\left( 1-\frac{\tau}{T} \right)T}\#\left( 16 \right) \end{aligned}$$

Thus we can treat a lag by multiplying the exponential growth rate by the factor (1$-\tau/T$). This is not an exact solution, but simulations of the two-species Lotka-Volterra competition model over a wide range of parameters showed that it had a mean error of 0.08 and median error of 0.008. (Error is defined as the difference between the fixed point of the model and the true fixed point, where such fixed points are defined as relative fractions with a maximum of one.)

The success of the approximation indicates that the effect of a time lag on growth rate is often predictable. A species with a high exponential growth rate and a significant time lag will behave more like a species with a lower exponential rate and no lag. One example of this effect can be seen in the performance of species *Pa*. Its exponential growth rate is similar to that of *Pci* and *Ea* (Supplementary Figure 5a), but its time lag is significant (Supplementary Figure 5e-f), causing its modified growth rate to be less (Supplementary Figure 5b). In competition, *Pa* dominates at low dilution factors, but loses to *Pci* and *Ea* as dilution increases (Supplementary Figure 1), indicating that they are the faster growers.

An anomalous scenario that cannot be explained with a time lag is the better performance of *Ea* over *Pp* at high dilution factors. The two species usually coexist at all dilution factors (Supplementary Figure 1), but the fraction of *Ea* increases with dilution, even though it is a slower grower with a shorter time lag (Supplementary Figure 5). Estimates of (threshold) growth rate are more similar for these two species (p = 0.08) than for other pairs of species (p < 0.001), however, indicating that the breakdown of the model’s predictions may have more to do with the imperfection of the model that its qualitative features. For example, the model assumes logistic growth, but this assumption could be wrong for many reasons; perhaps the growth rate fluctuates in time due to the changing concentration of the different carbon sources in the media (our media contains glucose, potassium citrate, and cholesterol; see Methods).

**Supplementary Note 4: Qualitative predictions of resource-explicit models recapitulate LV model’s qualitative predictions**

While the two-species LV competition model provided us with mathematical intuition and simple experimental predictions, other models also predict that increasing death favors fast growers.


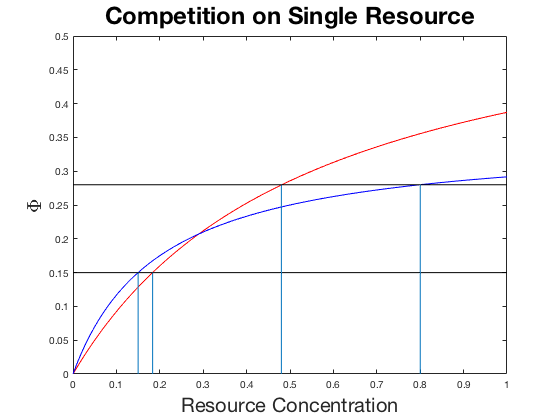


a_1_=0.6, b_1_=0.55

a_2_=0.35, b_2_=0.2

In resource-explicit models, species interact only through resource consumption. The simplest form of such a model involves one resource and two species in either a chemostat environment with continuous dilutions, or a batch culture with discrete dilutions (Stewart & Levin, 1973). The continuous model has a dilution rate $\rho$ equal to the incoming rate of fresh resources:

**Supplementary Figure 12:** A tradeoff between maximal growth rate *a* and half-maximum constant *b* can cause the winner of a competition to reverse as dilution increases.

$$\begin{aligned} \dot{N_{i}}=N_{i}\left( \frac{\phi_{i}}{e_{i}}-\rho\right)\#\left( 17 \right) \end{aligned}$$

$$\begin{aligned} \dot{R}=\rho\left( c-R \right)-\sum_{i} N_{i}\phi_{i}\#\left( 18 \right) \end{aligned}$$

$$\begin{aligned} \phi_{i}=\frac{a_{i}R}{R+b_{i}}\#\left( 19 \right) \end{aligned}$$

where $N_{i}$ are the species concentrations, $R$ is the resource concentration, $c$is initial resource concentration, and $e_{i}$ is the amount of resource needed for species $i$to reproduce. The resource uptake functions $\phi_{i}$ have a Michaelis-Menten form, with maximal growth rates $a_{i}$ and half-maximum constants $b_{i}$.

The winner of the competition can be determined by setting the per-capita growth rate to zero. The species with the lower $R^{*}$ excludes the other species, because it has a higher per-capita growth rate at a lower resource concentration. Depending on the amount of dilution $\rho$, the winner of the competition can change. Such a reversal requires a tradeoff in the resource uptake functions between $a_{i}$ and $b_{i}$. Neither stable coexistence nor bistability are allowed in this model.

The discrete model is formed by setting $\rho=0$, because there is no flow into or out of the system:

$$\begin{aligned} \dot{N_{i}}=N_{i}\left( \frac{\phi_{i}}{e_{i}} \right)\#\left( 20 \right) \end{aligned}$$

$$\begin{aligned} \dot{R}=\sum_{i} N_{i}\phi_{i}\#\left( 21 \right) \end{aligned}$$

At the end of each dilution cycle, the resource concentration is re-set to $c$, and the $N_{i}$ are multiplied by a dilution fraction $d$.

The surprising result of the discrete model is that its temporal fluctuations allow stable coexistence and bistability:

­­

**Supplementary Figure 13:** Two cases of the daily dilution model are shown: on the left, $e_{1} = e_{2} = 1$; on the right, $e_{1} = 0.7$, $e_{2} = 1$. In both plots, $a_{1} = 10$, $a_{2} = 1$, $b_{1} = 5$, $b_{2} = 0.05$. Dilution factor increases as dilution fraction decreases.

The model can be expanded to include more than one resource. This adds complexity to the model, but a simple graphical method makes it possible to determine the dominant species at a particular dilution rate and initial resource concentration (Tilman, 1982). Coexistence and bistability are possible in both the continuous and discrete versions of the two- (or more) resource model.

**Supplementary References**

1. Fox, J. W. (2013). The intermediate disturbance hypothesis should be abandoned. Trends in ecology & evolution, 28(2), 86-92.
2. Stewart, F. M., & Levin, B. R. (1973). Partitioning of resources and the outcome of interspecific competition: a model and some general considerations. *The American Naturalist*, *107*(954), 171-198.
3. Tilman, D. (1982). *Resource competition and community structure*. Princeton university press.
