# Supplementary material for: Mortality causes universal changes in microbial community composition
Source: Nat Commun. 2019 May 9;10:2120. doi: 10.1038/s41467-019-09925-0 (PMC6509412; doi:10.1038/s41467-019-09925-0)
Supplement: Supplementary file 3 — Reporting Summary [file 41467_2019_9925_MOESM3_ESM.pdf]

nature research

Corresponding author(s): Jeff Gore

Last updated by author(s): Dec 29, 2018

Reporting Summary

Nature Research wishes to improve the reproducibility of the work that we publish. This form provides structure for consistency and transparency in reporting. For further information on Nature Research policies, see Authors & Referees and the Editorial Policy Checklist.

Statistics

For all statistical analyses, confirm that the following items are present in the figure legend, table legend, main text, or Methods section.

n/a	Confirmed	
☐	☒	The exact sample size (*n*) for each experimental group/condition, given as a discrete number and unit of measurement
☐	☒	A statement on whether measurements were taken from distinct samples or whether the same sample was measured repeatedly
☐	☒	The statistical test(s) used AND whether they are one- or two-sided<br>*Only common tests should be described solely by name; describe more complex techniques in the Methods section.*
☒	☐	A description of all covariates tested
☐	☒	A description of any assumptions or corrections, such as tests of normality and adjustment for multiple comparisons
☐	☒	A full description of the statistical parameters including central tendency (e.g. means) or other basic estimates (e.g. regression coefficient) AND variation (e.g. standard deviation) or associated estimates of uncertainty (e.g. confidence intervals)
☐	☒	For null hypothesis testing, the test statistic (e.g. *F*, *t*, *r*) with confidence intervals, effect sizes, degrees of freedom and *P* value noted<br>*Give P values as exact values whenever suitable.*
☒	☐	For Bayesian analysis, information on the choice of priors and Markov chain Monte Carlo settings
☒	☐	For hierarchical and complex designs, identification of the appropriate level for tests and full reporting of outcomes
☒	☐	Estimates of effect sizes (e.g. Cohen's *d*, Pearson's *r*), indicating how they were calculated

Our web collection on statistics for biologists contains articles on many of the points above.

Software and code

Policy information about availability of computer code

Data collection	The Infinite M200 Pro Tecan Plate Reader, and proprietary software, was used to collect growth curve data, where OD 600 nm was measured. Microsoft Excel (v. 16.16.5) was used to collect and store colony-counting data. RStudio was used to import data from Excel and plot data (v. 1.1.383).
Data analysis	Python (v. 2.7.10) and MATLAB (R2016a) were used to analyze data (measuring growth rates, time lags, assembly rule prediction accuracy, etc.).

For manuscripts utilizing custom algorithms or software that are central to the research but not yet described in published literature, software must be made available to editors/reviewers. We strongly encourage code deposition in a community repository (e.g. GitHub). See the Nature Research guidelines for submitting code & software for further information.

Data

Policy information about availability of data

All manuscripts must include a data availability statement. This statement should provide the following information, where applicable:

- Accession codes, unique identifiers, or web links for publicly available datasets
- A list of figures that have associated raw data
- A description of any restrictions on data availability

The source data underlying Figs 1b, 3b, f, 4a-c, and Supplementary Figs 1, 2, 5 and 7 are provided as a Source Data file. Access to the data is also publicly available at https://figshare.com/projects/Added_mortality_causes_universal_changes_in_microbial_community_composition/58304. A reporting summary for this Article is available as a Supplementary Information file.

Field-specific reporting

Please select the one below that is the best fit for your research. If you are not sure, read the appropriate sections before making your selection.

☐ Life sciences ☐ Behavioural & social sciences ☒ Ecological, evolutionary & environmental sciences

For a reference copy of the document with all sections, see nature.com/documents/nr-reporting-summary-flat.pdf

Ecological, evolutionary & environmental sciences study design

All studies must disclose on these points even when the disclosure is negative.

Study description

Five species of soil bacteria were cocultured in environments with added mortality to determine how coculture outcomes were affected by mortality. Outcomes were classified as a) dominance of one species, b) coexistence of two or more species, c) bistability of multiple outcomes, where the final state depended upon initial fractions of species. Cocultures were grown in 96-well plates and diluted on a daily basis into fresh medium, over a range of daily dilution factors from 10 to 10^6. Monocultures were reproduced at least 10 times and pairwise cocultures were reproduced at least twice, per experimental condition and starting fraction.

Research sample

The soil bacterial species used in this study were Enterobacter aerogenes (Ea, ATCC#13048), Pseudomonas aurantiaca (Pa, ATCC#33663), Pseudomonas citronellolis (Pci, ATCC#13674), Pseudomonas putida (Pp, ATCC#12633) and Pseudomonas veronii (Pv, ATCC#700474). All species were obtained from ATCC. These species were chosen because of their availability in the laboratory, and knowledge gained from their usage in previous studies by other members of the laboratory.

Sampling strategy

Population sampling was performed by plating diluted cultures onto Petri dishes containing nutrient agar. The cultures were diluted to a sufficiently low density so that individual cells could grow into distinct colonies, and the number of colonies could be multiplied by the dilution factor to determine the population size. The mean number of colonies, per plate, per experimental condition, was 42. We were interested in relative abundances more than absolute abundances, and thus errors of relative fractions. For a single sampling from a plate of 42 colonies with two species, a 0.5 fraction has an error of 0.07 (according to our estimate using the SD of the beta distribution-- see Methods). One colony out of 42 has an error of 0.03. For this reason, we assigned an extinction threshold of 3% to distinguish between dominance of one species and coexistence of two species.

Data collection

First and third authors carried out all experiments with two or more species, and collected data by counting colonies to determine relative fractions, as explained above. The first author collected data from one-species monoculture experiments to determine growth rates and carrying capacities.

Timing and spatial scale

Data collection began in July 2016 and proceeded until August 2018. In the fall of 2017, we switched to a minimal and defined growth medium, after realizing that the complex and undefined medium caused quantitative differences in results from batch to batch, even when using the same brand and type of product (we realized this then, because we had just prepared a new batch of concentrated medium for the first time). All experiments were re-done in the defined and minimal medium. All experiments consisted of five to seven 24-hour dilution cycles. New data was collected in November and December of 2018, in order to address questions from manuscript reviewers. This data consisted of monoculture measurements (growth rates, carrying capacities).

Data exclusions

We excluded data from experiments in which contamination occurred (e.g. species invaded from other wells) due to its inaccuracy. We also excluded data obtained from the new batch of the complex medium, mentioned above, which gave quantitatively different results. Data from the complex medium was included in the supplementary section but not included in the main text; we showed data only from the minimal and defined medium in the main text. We emphasized the data from the defined medium because we want readers to be able to reproduce our results, but we included the data from the complex medium in order to show that the phenomenon is reproducible in complex medium, and that quantitative predictions can be made assuming one uses the same batch of complex medium.

Reproducibility

All monoculture experiments to measure growth rate were repeated at least 10 times. Ordering of growth rates was a crucial question in our study, so we used two methods for calculating growth rate (see Figs. S3, S4, S5) and verified that the order was the same with both methods. All two-species experiments were reproduced at least twice per experimental condition. Two- and three-species results highlighted in the main text were reproduced at least three times per experimental condition. In two- and three-species experiments, results differed quantitatively, but there were few qualitative differences. When the latter did occur, it was a borderline case (e.g. a species survived in coculture over some range of dilution factors, and the edge of the range moved slightly between experiments-- see Fig. S8).

Randomization

All experiments began from the same frozen stocks of individual species. Individual species were streaked out from these frozen stocks on Petri dishes, where single colonies grew. Each competition experiment began with single colonies picked of each species. Each species was grown separately in liquid growth medium for two 24-hour dilution cycles before beginning competition, and the culture was stirred vigorously before diluting into the experimental plate, to ensure that competition began identically from different biological replicates (colonies).

Blinding

The first and third authors performed coculture experiments and collected data separately, to ensure that both arrived at the same conclusions separately.

Did the study involve field work? ☐ Yes ☒ No

Reporting for specific materials, systems and methods

We require information from authors about some types of materials, experimental systems and methods used in many studies. Here, indicate whether each material, system or method listed is relevant to your study. If you are not sure if a list item applies to your research, read the appropriate section before selecting a response.

Materials & experimental systems

n/a	Involved in the study
☒	☐ Antibodies
☒	☐ Eukaryotic cell lines
☒	☐ Palaeontology
☒	☐ Animals and other organisms
☒	☐ Human research participants
☒	☐ Clinical data

Methods

n/a	Involved in the study
☒	☐ ChIP-seq
☒	☐ Flow cytometry
☒	☐ MRI-based neuroimaging
